# Molecular Docking and Dynamics Investigations for Identifying Potential Inhibitors of the 3-Chymotrypsin-like Protease of SARS-CoV-2: Repurposing of Approved Pyrimidonic Pharmaceuticals for COVID-19 Treatment

**DOI:** 10.3390/molecules26247458

**Published:** 2021-12-09

**Authors:** Amin Osman Elzupir

**Affiliations:** College of Science, Deanship of Scientific Research, Imam Mohammad Ibn Saud Islamic University (IMSIU), Riyadh 11623, Saudi Arabia; aoalamalhuda@imamu.edu.sa

**Keywords:** coronavirus SARS-CoV-2, COVID-19, 3-chymotrypsin-like protease, pyrimidonic pharmaceuticals, molecular dynamics simulations, binding free energy

## Abstract

This study demonstrates the inhibitory effect of 42 pyrimidonic pharmaceuticals (PPs) on the 3-chymotrypsin-like protease of SARS-CoV-2 (3CL^pro^) through molecular docking, molecular dynamics simulations, and free binding energies by means of molecular mechanics–Poisson Boltzmann surface area (MM-PBSA) and molecular mechanics–generalized Born surface area (MM-GBSA). Of these tested PPs, 11 drugs approved by the US Food and Drug Administration showed an excellent binding affinity to the catalytic residues of 3CL^pro^ of His41 and Cys145: uracil mustard, cytarabine, floxuridine, trifluridine, stavudine, lamivudine, zalcitabine, telbivudine, tipiracil, citicoline, and uridine triacetate. Their percentage of residues involved in binding at the active sites ranged from 56 to 100, and their binding affinities were in the range from −4.6 ± 0.14 to −7.0 ± 0.19 kcal/mol. The molecular dynamics as determined by a 200 ns simulation run of solvated docked complexes confirmed the stability of PP conformations that bound to the catalytic dyad and the active sites of 3CL^pro^. The free energy of binding also demonstrates the stability of the PP–3CL^pro^ complexes. Citicoline and uridine triacetate showed free binding energies of −25.53 and −7.07 kcal/mol, respectively. Therefore, I recommend that they be repurposed for the fight against COVID-19, following proper experimental and clinical validation.

## 1. Introduction

Over a year has passed since the COVID-19 pandemic began. Some vaccines, such as those by Pfizer and Moderna, and some drugs, such as remdesivir, have been approved for use in therapy. The efforts by governments, health organizations, and other sectors to stem the alarmingly increasing numbers of deaths and cases were unprecedented [1,2,3,4,5,6]. However, SARS-CoV-2 continues to threaten the world, with over four million deaths and 227 million cases as of 16 September 2021 (https://www.worldometers.info/coronavirus/ accessed on 27 November 2021). COVID-19 was declared a pandemic by the World Health Organization on 11 March 2020. Today, the new SARS-CoV-2 virus, the causative agent of COVID-19, has been detected in almost every country on the planet [5,7,8,9].

Coronaviruses are positive-stranded RNA viruses with the largest viral genomes ever known, ranging from 16 to 32 kb. The 3-chymotrypsin-like protease (3CL^pro^) produced by SARS-CoV-2 is a cysteine protease encoded as nonstructural protein 3 in the polyprotein. 3CL^pro^ is responsible for the cleavage of 11 specific sites of polyproteins (pp1a, pp1ab) produced by the 229E gene. These polyproteins are involved in the production of a functional polypeptide essential for viral replication and transcription. Further, the specificity of 3CL^pro^ is dissimilar to that of human host-cell protease. Thus, 3CL^pro^ has become the focus of drug repurposing and development programs to combat the COVID-19 pandemic [10,11,12,13].

Recent and ongoing research has reported that some pharmaceutical, synthetic, and natural products can act as 3CL^pro^ inhibitors or against SARS-CoV-2 in general. These include selenium-containing heterocyclic compounds, chloroquine phosphate, indinavir, darunavir, lopinavir, eravacycline, naproxen, salix cortex, antioxidants, chiral phytochemicals from *Opuntia ficus-indica*, elbasvir, valrubicin, favipiravir isoflavone, and myricitrin [6,14,15,16,17,18,19,20,21,22]. Although some of these have entered human clinical trials or were even approved, more studies are still needed. The importance of the pyridone ring was highlighted in synthetic materials and drugs containing pyridone [11,23]. The pyrimidone ring has the exact shape of pyridine but is more functionalized and electron-deficient. Herein, we screened the inhibitory activity of 42 approved pyrimidonic pharmaceuticals (PPs) against 3CL^pro^ using a combination of molecular docking analyses, molecular dynamics simulations, and calculations of the MM-PBSA and MM-GBSA binding free energies. The sites of action of active inhibitors were investigated, discussed, and explored.

## 2. Materials and Methods

### 2.1. The Pyrimidonic Pharmaceuticals (PPs)

The PPs were selected using the search engine of the drug bank database. The search uncovered 46 PPs; the pharmaceuticals containing caffeine were entirely excluded as all except enprofylline have previously been studied. In addition, the macropolymeric drug mipomersen, drugs composed of a mixture of drugs, and withdrawn drugs were not included in this study. The chosen drugs were classified into four categories according to their structures. 1PPs have only one heterocycle, 2aPPs have two, 2bPPs have two heterocycles with a pyrimidone ring having an extra carbonyl group, and 3PPs have three or more heterocycles (Table 1).

### 2.2. Generation and Energy Minimization of the PPs and 3CL^pro^

The 3D structures of the selected PPs were downloaded from the PubChem website as SDF files; their energy was minimized for 10,000 steepest descent steps at 5000 conjugate gradient steps using antechamber plugin UCSF Chimera [24,25]. For alogliptin, the 3D structure was obtained by utilizing OpenBabel converter tools and ChemSkech [26]. The crystal structure of SARS-CoV-2 3CL^pro^ was obtained from the Protein Data Bank database website (PDB ID: 6Y2E). For analysis, water was removed from the 3CL^pro^ structure, and the energy was then minimized for 1000 steepest descent steps at 20 conjugate gradient steps.

### 2.3. Molecular Docking

Blind molecular docking experiments were performed using the AutoDock Vina tool implemented with the interactive visualization and analysis program UCSF Chimera. The default parameter values were adopted with a grid box (−15 × −25  × 15) Å, centered at (35, 65, 65) Å. The predicted affinity values of the score were observed using the View Dock tool. The binding between ligands and 3CL^pro^ active sites and the images were processed and visualized using UCSF Chimera [24,25,26,27,28].

### 2.4. Molecular Dynamics Simulations

MD simulations were performed as previously described [29]. The PP ligands were separated from the docked complexes using UCSF Chimera. The missed hydrogens were added and saved as PDB files using AMBER’s large-structure serial numbering. Topology files and parameters of the receptor and the ligands were made using leap and antechamber of Amber Tools 21 [30,30], utilizing Amber force fields of GAFF2 [31] and ff14SB [32] to assign inhibitors and 3CL^pro^ structure, respectively. The systems were solvated with TIP3P water molecules [33] and were neutralized via sodium ions. Subsequently, molecular dynamics (MD) simulations were performed by means of the Nanoscale Molecular Dynamics (NAMD) Simulation 2.6 program [34]. Each system was minimized for 1 ps at 273.15 K using the NVE ensemble. The temperature was gradually increased to 310 K using the NVT ensemble in a protocol consisting of 1600 minimization steps. Then, each system was minimized for 10 ps at 310 K followed by 200 ns of MD simulation control using the NVT ensemble at 310 K and a time step of 2 fs. In order to calculate electrostatic interactions, the particle mesh Ewald process and periodic boundary conditions were applied [35,36]. The root-mean-square fluctuation (RMSF) and the root-mean-square deviation (RMSD) for each system were obtained by analyzing the trajectory using the VMD 1.8 program [37].

### 2.5. The Binding Free Energies

The binding free energies of the PP-3CL^pro^ complexes were calculated by means of molecular mechanics–Poisson Boltzmann surface area (MM-PBSA) and molecular mechanics–generalized Born surface area (MM-GBSA) using the MMPBSA.py module of Amber Tools 21 [38]. The MD simulation over 200 ns provided several conformations sampled after equilibrium, using the last frames to lessen the computational cost. CPPTRAJ was used to obtain the snapshots [39]. The conformational changes were evaluated through quasi-harmonic entropy approximation [40]. The free energy of the binding interaction between inhibitors and 3CL^pro^ complexes can be obtained via the following equations:

ΔG = ΔH − TΔS(1)

ΔH = ΔG_gas_ + ΔG_sol_ (2)

ΔG_gas_ = E_vdw_ + E_elec_ (3)

ΔG_sol_ = E_pb/gb_ + E_np_ (4)

where ΔH represents enthalpy change, TΔS represents the entropic contribution, E_vdw_ represents the van der Waals interaction energy, E_ele_ represents the electrostatic interaction energy, ΔG_sol_ represents the polar solvation energy, and E_np_ represents the nonpolar solvation energy.

## 3. Results and Discussion

**Figure 1:**
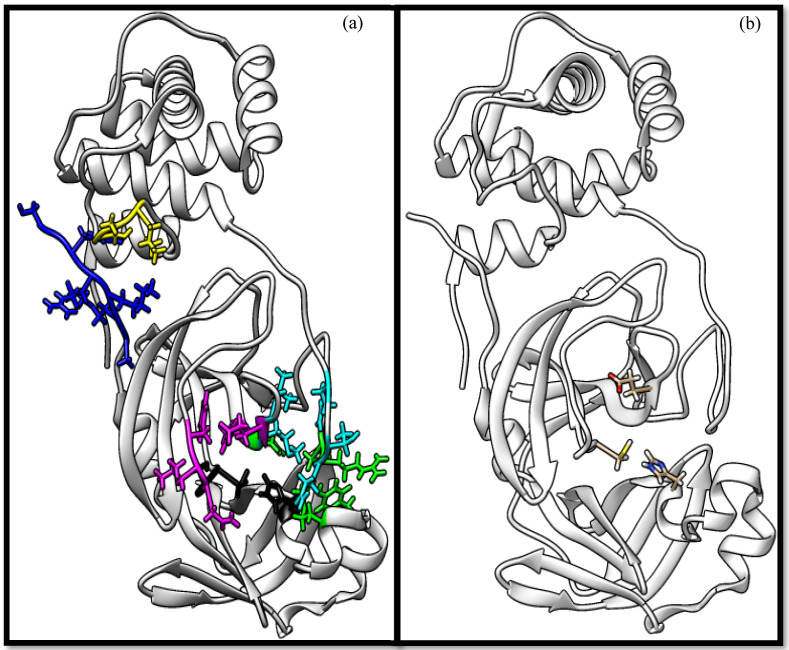
The crystal structure of chymotrypsin-like protease of SARS-CoV-2 (PDB ID: 6Y2E) and its active residues. (**a**) Color indicates the residues involved in the formation of the S1 site (shown in magenta), S1 site from the other promotor (blue), S2 site (green), S4 site (cyan), and S1’ site (black), in addition to SER 284, ALA 285, and LEU 286 (yellow). (**b**) Only the catalytic dyad and GLU 166 residues.

The results of the molecular docking are tabulated in Tables S1–S4. Figure 1 shows the catalytic dyad and the active sites of 3CL^pro^. The crucial residues HIS 41, GLY 143, SER 144, and CYS 145 forming the S1′ site are shown in black. Then, PHE 140, LUE 141, ASN 142, HIS 163, GLU 166 (magenta), and the *N*-terminal amino acid residues (blue) are involved in the formation of the S1 subsite of the substrate-binding pocket. The MET 49, TYR 54, HIS 164, ASP 187, and ARG R188 residues form the S2 site (green). MET 165, LEU 167, GLN 189, THR 190, and GLN 192 comprise the S4 site (cyan). The SER 284, ALA 285, and LEU 286 residues (yellow) are a result of genetic mutation leading to an increase in the SASR-CoV-2 3CL^pro^ activity of 3.6 fold over that of the 3CL^pro^ predecessor of SARS-CoV [12,41].

### 3.1. Molecular Docking

**Figure 2:**
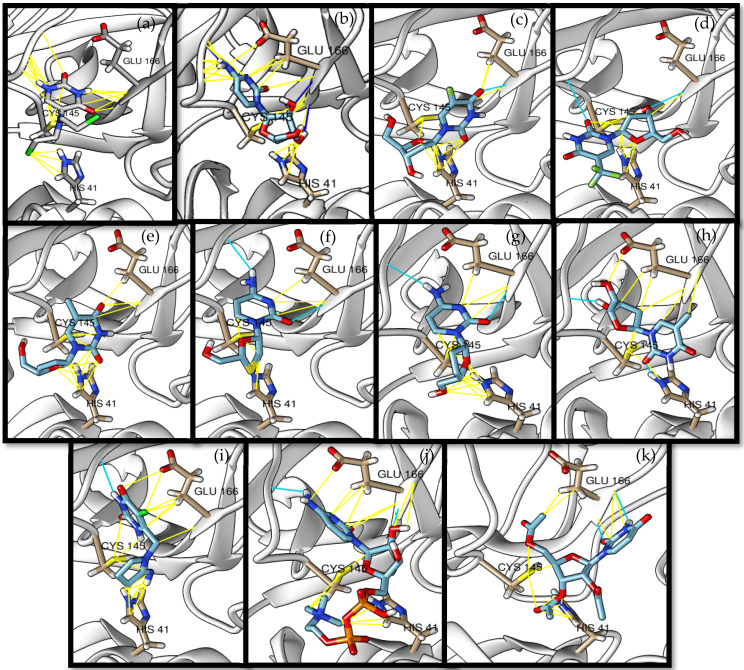
The PPs docked with 3CL^pro^, focusing on contacts with HIS 41, CYS 145, and GLU 166. (**a**) uracil mustard, (**b**) cytarabine, (**c**) floxuridine, (**d**) trifluridine, (**e**) stavudine, (**f**) lamivudine, (**g**) zalcitabine, (**h**) telbivudine, (**i**) tipiracil, (**j**) citicoline, (**k**) uridine triacetate. The hydrocarbon skeleton is shown in cyan, nitrogen atoms are blue, and oxygens are red. Hydrogen bonds are represented by blue lines; van der Waals forces are represented in yellow.

The docked complexes of the top 11 candidates are depicted in Figure 2. Their binding affinities to the active sites of 3CL^pro^ are shown in Table 2. The 3PPs showed significant interactions with the residues LEU 286, SER 284, and ALA 285, and a relatively lower interaction ratio to the catalytic dyad, in contrast to the other groups. Of the 3PPs, alogliptin and flavin mononucleotide were found to have the highest binding percentage with the catalytic dyad and to form hydrogen bonds with the S1 and S’1 sites. These were followed by riboflavin and sofosbuvir with an advantage in binding to the LEU 286 residue (Table S1). Flavin adenine dinucleotide showed excellent binding affinity to LEU 286 but not with the catalytic dyad. Zidovudine and gemcitabine demonstrated similar activity to alogliptin and flavin mononucleotide (Tables S2 and S3).

Among the 2bPPs, anti-hepatitis B infection telbivudine, anti-orotic aciduria uridine triacetate, and anticancer tipiracil were found to have the highest binding to 3CL^pro^ active sites, followed by antimetabolite floxuridine, anti-herpesvirus trifluridine, and anti-HIV stavudine. Here, it is worth noting the importance of the molecular structure, as this set differed from the previous one by its increased ability to bind to the 3CL^pro^ catalytic dyad. The 2aPPs showed similar activity to that of the 2bPPs. Anticancer cytarabine, anti-glaucoma citicoline, and anti-HIV drugs lamivudine and zalcitabine showed promising inhibitory activity (Table S3).

Finally, but very importantly, of the 1PPs, the chemotherapy drug uracil mustard showed binding to the catalytic dyad with all of its simulated conformations, followed by anti-cytomegalovirus cidofovir (Table S4).

### 3.2. Molecular Dynamics Simulations

**Figure 3:**
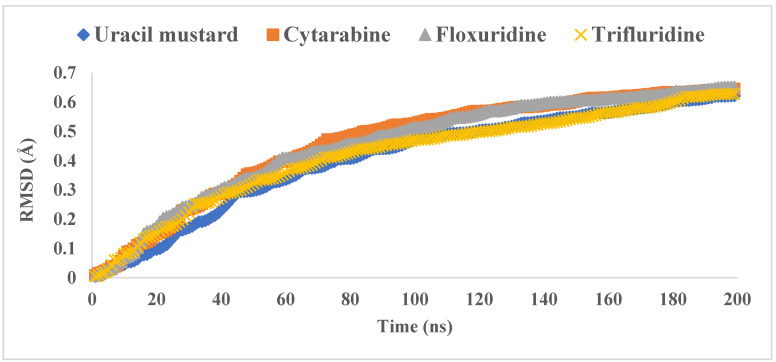

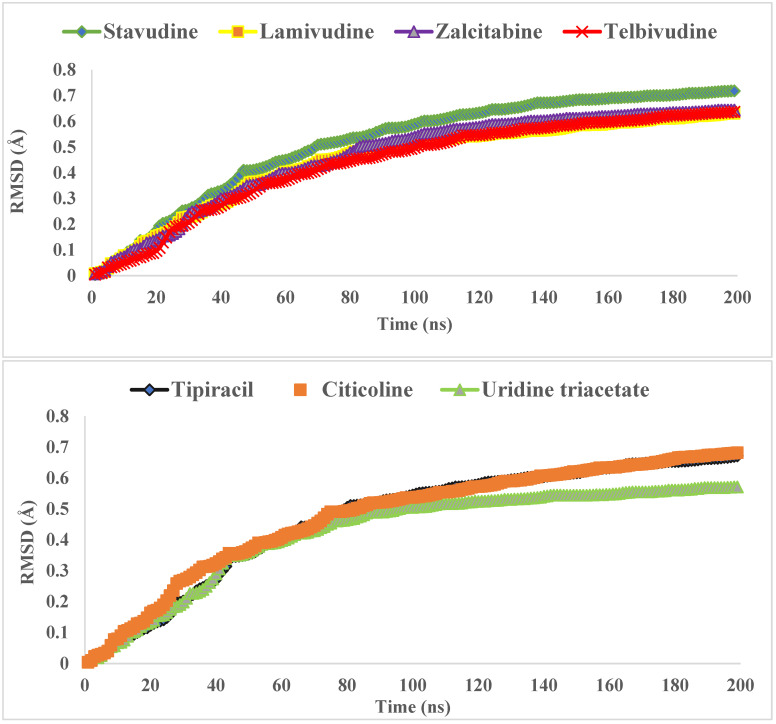
The RMSD values of the simulated PP-3CL^pro^ complexes throughout the 200 ns production runs.

**Figure 4:**
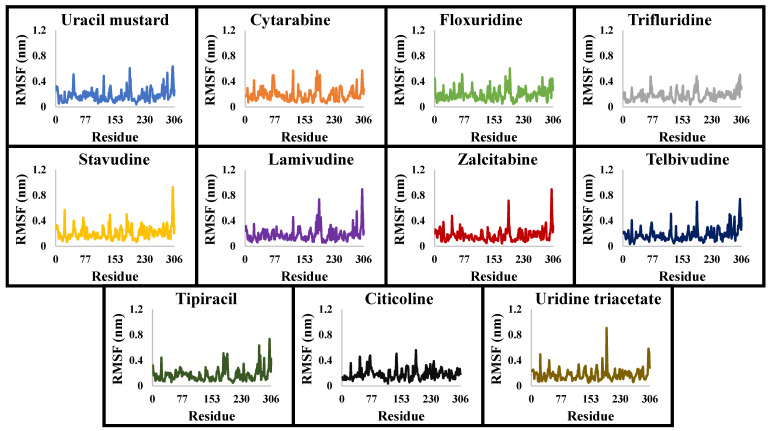
The RMSF values of the simulated PP-3CL^pro^ complexes throughout the 200 ns production runs.

MD was performed on the hole complexes of the top 11 PPs candidates. Based on the conformer score energy from docking, the complex with the conformer with the lowest value and interacting with the 3CL^pro^ active site was selected. The RMSDs were computed along the trajectories using the initial structure as a reference. Figure 3 shows that the binding of PPs significantly affected the equilibration states of 3CL^pro^, as the majority of the tested systems reached their equilibrium at around 100 ns. The PP-3CLpro complexes revealed relatively lower average values for the RMSDs, between 0.41 and 0.52 Å, throughout the simulation, clarifying their good behavior in forming stable complexes. Moreover, the fluctuations in the 3CL^pro^ backbone residues were analyzed by means of the RMSF (Figure 4). The 3CL^pro^/PPs exhibited lower fluctuations, particularly at the active site. The fluctuations at the catalytic dyad and GLU 166 were minor, demonstrating the loss of flexibility at these regions upon binding to the PPs. Table 3 shows the superior stability of the PP-3CL^pro^ complexes formed throughout the production runs; these results support the use of these PPs as 3CL^pro^ inhibitors.

### 3.3. The Binding Free Energies

The data on the binding free energies of the PP-3CL^pro^ complexes are tabulated in Table 4. In both MM-GBSA and MM-PBSA, van der Waals and electrostatic interactions acted as driving forces for the PP ligands to bind to 3CL^pro^, contrasting the solvation energies. The MM-GBSA and MM-PBSA results suggest that the PPs have an excellent ability to inhibit 3CL^pro^. Of these PPs, citicoline revealed the most promising inhibitory activity, followed by uridine triacetate (Table 4).

Among the challenges of discovering 3CL^pro^ inhibitors for COVID-19 treatment, these inhibitors must be highly bioavailable inside the cytosol [13]. The 2aPP and 2bPP structures contain a primidone heterocycle and ribose ring. These heterocycles increase their hydrophilicity and solubility in plasma. Thus, they can satisfy the requirement of bioavailability. Citicoline, the most promising inhibitor among the PPs investigated, has high hydrophilicity and good ADME properties [42,43]. The 2aPPs and 2bPPs also have an intermediate structure size among the PP groups. This sheds light on the importance of the size and general structural features of PPs acting as 3CL^pro^ inhibitors.

Recent reports suggest a general hypothesis that 3CL^pro^ inhibitors comprise electrophilic sites such as Michael acceptors [12]. That the pyrimidone ring is highly electron-deficient clarifies and confirms this hypothesis. The pyrimidone ring plays an essential role in PPs’ inhibitory activity and has a high tendency to form hydrogen bonds, particularly with the GLU 166 residue. This may preclude the formation of the S1 pocket. Contacts between the pyrimidone ring and HIS 41 were observed in floxuridine, stavudine, and telbivudine. In all these cases, HIS 41 interacts with the oxygen of the pyrimidone group. Further, the electrophilic carbon, nitrogen, and oxygen in the pyrimidone ring were attracted to bind with the sulfur of the CYS 145 residue.

Interestingly, most PPs investigated were previously studied against SARS-CoV-2. For example, flavin mononucleotide and flavin adenine dinucleotide have been suggested as good 3CL^pro^ and RNA-dependent RNA polymerase inhibitors, respectively [22,44]. Riboflavin and sofosbuvir were shown to be suitable inhibitors of the spike protein S1 domain/ACE2 and RNA-dependent RNA polymerase [45,46,47]. Alogliptin was also suggested as a 3CL^pro^ inhibitor; however, the enzymatic assay demonstrated its inactivity against 3CL^pro^ [48]. Compelling clues have been found regarding the use of zidovudine and gemcitabine against spike protein/human ACE2 and in the inhibition SARS-CoV-2 in cell culture [49,50,51,52,53]. Gemcitabine and cidofovir were reported to inhibit SARS-CoV and SARS-CoV-2 proteins with IC_50_ values of 4.95 μM and 36 μM [54,55]. Further, telbivudine, tipiracil, cytarabine, and citicoline were recommended as 3CL^pro^ inhibitors [56,57,58,59,60,61]. This transitory literature scanning confirms these pharmaceuticals’ activity against 3CL^pro^ of SARS-CoV-2, as demonstrated in the present study.

To conclude, the inhibitory effect on 3CL^pro^ by PPs was investigated based on their ability to form hydrogen bonds and van der Waals interactions with the 3CL^pro^ active side through molecular docking, MD simulations, and the calculation of binding free energy. The overall analysis revealed 11 candidates from the initial set of 42 investigated PPs are promising 3CL^pro^ inhibitors. These include citicoline and uridine triacetate as the best choices, followed by telbivudine, trifluridine, lamivudine, cytarabine, stavudine, zalcitabine, tipiracil, floxuridine, and flavin mononucleotide. The interactions of PPs with the catalytic dyad and the active sides of 3CL^pro^ of SARS-CoV-2 were comprehensively and thoroughly investigated. The pyrimidone ring was found to play an essential role in the PPs’ inhibitory activity.

## Figures and Tables

**Table 1:** Structures of pyrimidonic pharmaceuticals and their classification according to the number of rings.

1PPs	2aPPs	2bPPs	3PPs
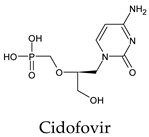	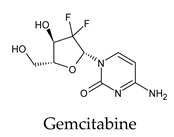	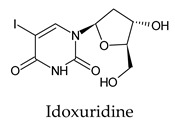	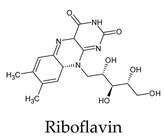
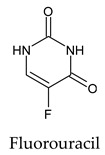	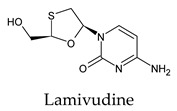	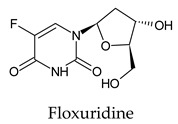	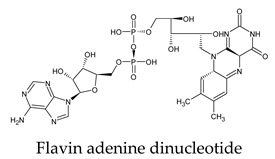
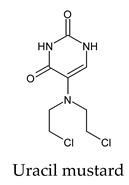	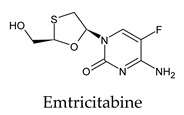	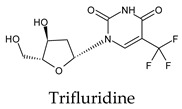	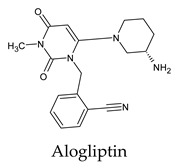
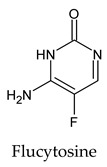	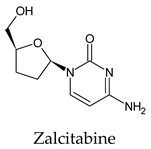	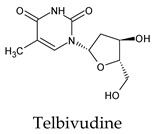	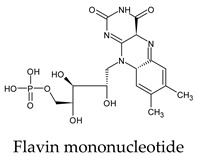
-	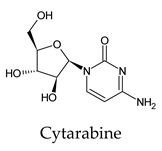	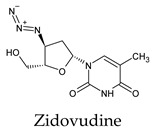	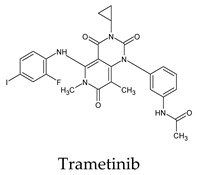
-	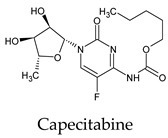	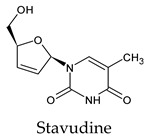	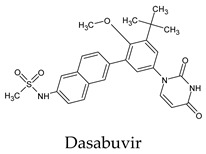
-	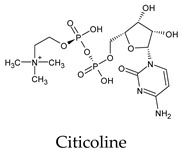	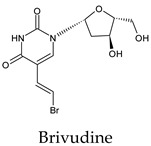	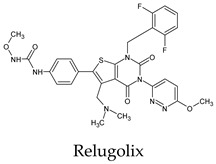
-	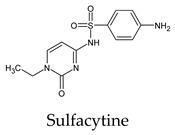	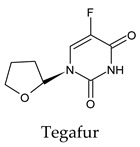	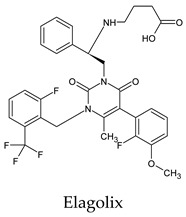
-	-	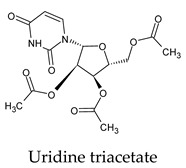	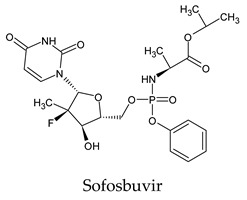
-	-	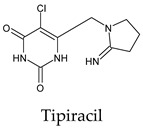	-
-	-	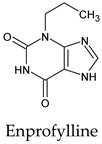	-

**Table 2:** The binding affinities of the potential pyrimidonic pharmaceuticals with 3-chymotrypsin-like protease (3CL^pro^).

Pharmaceutical Name	Binding Percentage ^a^	Score ± SD (kcal/mol) ^b^	RMSD	Hydrogen Bond (Number of Bonds/Number of Conformations)	Van Der Waals (Distance) (Number of Bonds/Number of Conformations)
Uracil mustard	a. 100 * c. 89 d. 100 e. 100 All. 100	a. −4.6 ± 0.14 c. −4.6 ± 0.14 d. −4.6 ± 0.14 e. −4.6 ± 0.14	a. 0.00–7.13 c. 0.00–7.13 d. 0.00–7.13 e. 0.00–7.13	a. HIS 163 (5/5), GLU 166 (3/3), LEU 141 (3/3), ASN 142 (1/1)	a. HIS 163 (31/5), GLU 166 (40/8), LEU 141 (19/5), ASN 142 (50/7), PHE 140 (8/5) c. MET 49 (52/7), HIS 164 (12/4) d. GLN 189 (32/9), MET 165 (28/8) e. HIS 41 (53/9), SER 144 (10/5), GLY 143 (4/1), CYS 145 (28/9)
Cytarabine	a. 67 c. 67 d. 67 e. 67 All. 67	a. −5.4 ± 0.26 c. −5.4 ± 0.26 d. −5.4 ± 0.26 e. −5.4 ± 0.26	a. 0.00–6.07 c. 0.00–6.07 d. 0.00–6.07 e. 0.00–6.07	a. GLU 166 (3/3), LEU 141 (2/2), HIS 163 (1/1), PHE 140 (2/2), ASN 142 (1/1) c. HIS 164 (2/2) d. GLN 189 (1/1) e. GLY 143 (1/1)	a. GLU 166 (44/5), LEU 141 (18/3), HIS 163 (18/4), PHE 140 (19/3), ASN 142 (27/6) c. HIS 164 (10/4), MET 49 (20/6) d. MET 165 (28/4), GLN 189 (4/2) e. GLY 143 (11/2), SER 144 (8/2), CYS 145 (15/6), HIS 41 (21/5)
Floxuridine	a. 44 c. 44 d. 44 e. 44 All. 56	a. 5.5 ± 0.13 c. 5.6 ± 0.25 d. 5.6 ± 0.25 e. 5.6 ± 0.19	a. 3.24–8.28 c. 0.00–8.28 d. 0.00–8.28 e. 0.00–8.28	a. ASN 142 (1/1), HIS 163 (2/2), GLU 166 (2/2), PHE 140 (1/1), LEU 141 (1/1) e. HIS 41 (1/1)	a. GLU 166 (23/4), LEU 141 (6/2), PHE 140 (8/2), HIS 163 (5/2), ASN 142 (19/4) c. MET 49 (9/2), HIS 164 (3/3) d. MET 165 (5/3), GLN 189 (4/1) e. GLY 143 (6/1), SER 144 (4/1), CYS 145 (5/3), HIS 41 (16/3)
Trifluridine	a. 44 c. 44 d. 44 e. 44 f. 11 All. 56	a. −6.03 ± 0.17 c. −6.03 ± 0.17 d. −6.03 ± 0.17 e. −6.03 ± 0.17 f. −5.7	a. 0.00–5.58 c. 0.00–5.58 d. 0.00–5.58 e. 0.00–5.58 f. 28.34–30.21	a. GLU 166 (2/2), ASN 142 (1/1). c. HIS 164 (2/2) e. GLY 143 (1/1)	a. GLU 166 (18/4), ASN 142 (15/3), HIS 163 (2/1), LEU 141 (2/1) b. c. HIS 164 (10/3), MET 49 (17/4) d. MET 165 (19/4), GLN 189 (1/1). e. CYS 145 (13/4), GLY 143 (10/2), HIS 41 (10/4) f. SER 284 (9/1)
Stavudine	a. 44 c. 56 d. 44 e. 56 All. 56	a. −5.6 ± 0.28 c. −5.6 ± 0.28 d. −5.6 ± 0.28 e. −5.6 ± 0.28	a. 27.12–32.34 c. 27.12–35.03 d. 27.12–32.34 e. 27.12–35.03	a. GLU 166 (1/1)	a. ASN 142 (13/3), GLU 166 (14/4), HIS 163 (2/1), LEU 141 (1/1) c. HIS 164 (6/3), MET 49 (20/5) d. MET 165 (12/4), GLN 189 (1/1) e. HIS 41 (39/5), GLY 143 (9/2), CYS 145 (7/3)
Lamivudine	a. 56 c. 44 d. 44 e. 56 f. 11 All. 67	a. −5.4 ± 0.24 c. −5.4 ± 0.28 d. −5.4 ± 0.25 e. −5.4 ± 0.24 f. −5.2	a. 0.00–4.77 c. 0.00–3.38 d. 0.00–4.77 e. 0.00–4.77 f. 26.28–28.62	a. HIS 163 (3/3), ASN 142 (1/1), PHE 140 (3/3), LEU 141 (2/2), GLU 166 (2/2) d. GLN 189 (1/1) e. SER 144 (2/2)	a. HIS 163 (26/5), ASN 142 (19/2), PHE 140 (31/5), LEU 141 (15/5), GLU 166 (46/5) c. MET 49 (12/4), HIS 164 (1/1) d. GLN 189 (12/2), MET 165 (10/4), LEU 167 (1/1) e. SER 144 (18/4), HIS 41 (5/2), CYS 145 (7/3) f. LEU 286 (1/1)
Zalcitabine	a. 44 b. 11 c. 33 d. 44 e. 44 f. 22 All. 67	a. −5.5 ± 0.29 b. −5.1 c. −5.4 ± 0.35 d. −5.5 ± 0.29 e. −5.5 ± 0.29 f. −5.1 ± 0.00	a. 0.00–6.39 b. 28.02–29.48 c. 0.00–6.39 d. 0.00–6.39 e. 0.00–6.39 f. 28.02–31.88	a. PHE 140 (3/3), LEU 141 (1/1), GLU 166 (3/3), ASN 142 (1/1) c. GLN 189 (1/1)	a. HIS 163 (15/3), PHE 140 (16/3), LEU 141 (9/3), GLU 166 (25/4), ASN 142 (11/3) b. LYS 5 (6/1), ARG 4 (12/1), PHE 3 (4/1) c. MET 49 (16/2), HIS 164 (5/3) d. GLN 189 (6/2), MET 165 (15/4) e. SER 144 (7/2), HIS 41 (13/2), CYS 145 (6/3) f. SER 284 (4/1), LEU 286 (4/1)
Telbivudine	a. 56 c. 44 d. 44 e. 56 f. 11 All. 67	a. −5.6 ± 0.38 c. −5.7 ± 0.42 d. −5.7 ± 0.42 e. −5.6 ± 0.38 f. −5.3	a. 0.00–7.50 c. 0.00–7.50 d. 0.00–7.50 e. 0.00–7.50 f. 22.85–24.17	a. ASN 142 (2/1), HIS 163 (3/3), GLU 166 (1/1), PHE 140 (2/2), LEU 141 (1/1) c. HIS 164 (1/1) d. GLN 189 (1/1) e. HIS 41 (1/1)	a. GLU 166 (36/4), HIS 163 (11/3), PHE 140 (11/3), ASN 142 (27/5), LEU 141 (9/3) c. HIS 164 (6/3), MET 49 (27/3) d. GLN 189 (10/3), MET 165 (14/4), e. CYS 145 (11/4), SER 144 (8/2), GLY 143 (3/1), HIS 41 (12/5) f. LEU 286 (5/1)
Tipiracil	a. 56 b. 11 c. 44 d. 44 e. 44 f. 11 All. 67	a. −5.8 ± 0.16 b. −5.7 c. −5.9 ± 0.17 d. −5.8 ± 0.08 e. −5.9 ± 0.17 f. −5.7	a. 26.28–29.69 b. 19.12–20.08 c. 26.28–29.69 d. 26.67–29.69 e. 26.28–29.69 f. 19.12–20.08	a. HIS 163 (2/2), GLU 166 (2/1), PHE 140 (2/2), ASN 142 (1/1) b. LYS 5 (1/1) c. HIS 164 (1/1) e. GLY 143 (1/1)	a. HIS 163 (14/3), GLU 166 (40/4), PHE 140 (8/3), ASN 142 (22/4), LEU 141 (10/3) b. PHE 3 (6/1), LYS 5 (9/1), ARG 4 (5/1) c. HIS 164 (8/4), MET 49 (22/3) d. MET 165 (9/3), GLN 189 (13/3) e. GLY 143 (7/2), HIS 41 (26/4), SER 144(2/1), CYS 145 (13/4) f. SER 284 (7/1), LEU 286 (2/1)
Citicoline	a. 56 * c. 56 d. 56 e. 56 All. 56	a. −7.0 ± 0.19 c. −7.0 ± 0.19 d. −7.0 ± 0.19 e. −7.0 ± 0.19	a. 0.00–8.01 c. 0.00–8.01 d. 0.00–8.01 e. 0.00–8.01	a. PHE 140 (2/2), GLU 166 (4/4), HIS 163 (3/3), ASN 142 (2/1), LEU 141 (1/1) e. SER 144 (1/1)	a. PHE 140 (24/5), GLU 166 (57/5), HIS 163 (21/5), ASN 142 (34/5), LEU 141 (21/5) c. MET 49 (23/5), HIS 164 (1/1) d. MET 165 (29/5), GLN 189 (6/3) e. SER 144 (12/3), GLY 143 (4/1), CYS 145 (9/5), HIS 41 (21/5)
Uridine triacetate	a. 56 c. 56 d. 56 e. 56 f. 11 All. 67	a. −6.2 ± 0.26 c. −6.2 ± 0.26 d. −6.2 ± 0.26 e. −6.2 ± 0.26 f. −6.4	a. 0.00–6.79 c. 0.00–6.79 d. 0.00–6.79 e. 0.00–6.79 f. 24.97–28.01	a. HIS 163 (2/2), GLU 166 (3/2) e. HIS 41 (1/1)	a. HIS 163 (5/2), GLU 166 (31/5), ASN 142 (30/5), PHE 140 (4/2), LEU 141 (6/2) c. MET 49 (18/5), HIS 164 (5/2) d. GLN 189 (15/4), MET 165 (18/4) e. CYS 145 (16/5), HIS 41 (35/5), SER 144 (4/2), GLY 143 (2/1) f. LEU 286 (1/1)

^a^ Binding percentage was calculated based on the number of conformations attached to the active sites of the CL^pro^ (nine conformations in total). ^b^ SD based on the other score energies of conformations. * Alphabetical order indicates the type of active site involved in bonding: a. S1 site, b. S1 site from the other promotor, c. S2 site, d. S4 site, e. S’1 site, and f. SER 284, ALA 285, and LEU 286 residues. When letters are missing, this means no interactions were observed at that site.

**Table 3:** The binding interactions of the potential pyrimidonic pharmaceuticals/3-chymotrypsin-like protease 3CL^pro^ complexes at different times throughout the production runs.

Pharmaceutical Name	100 ns	150 ns	200 ns
Uracil mustard	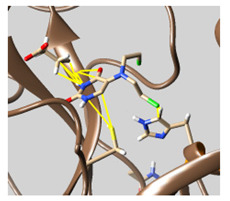	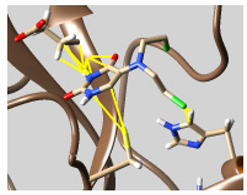	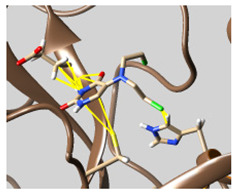
Cytarabine	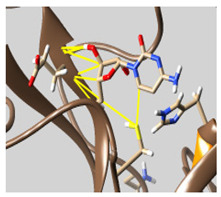	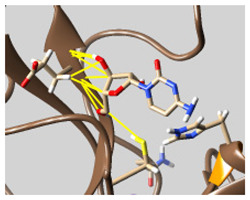	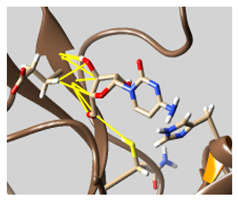
Floxuridine	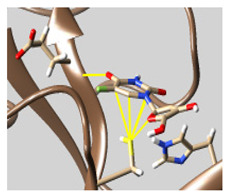	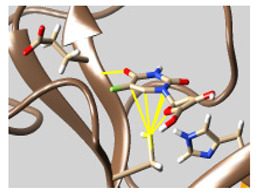	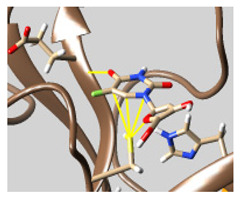
Trifluridine	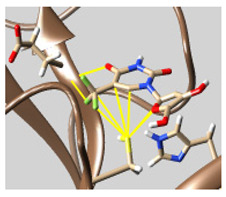	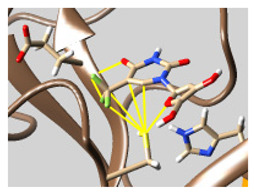	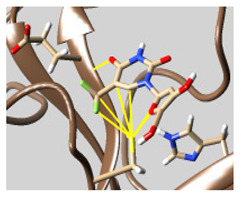
Stavudine	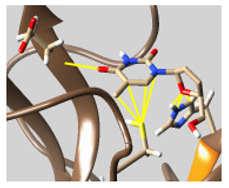	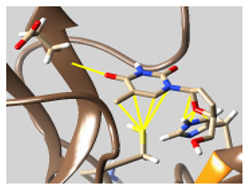	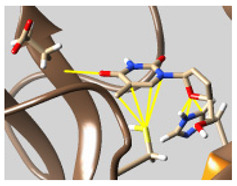
Lamivudine	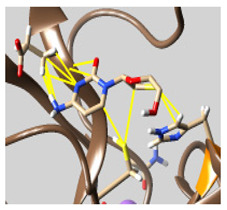	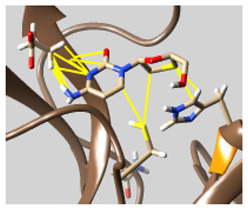	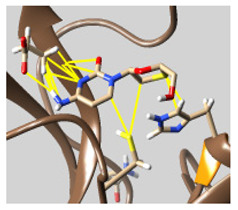
Zalcitabine	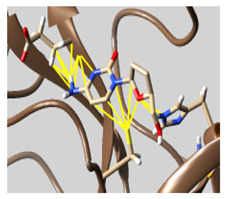	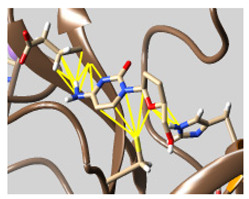	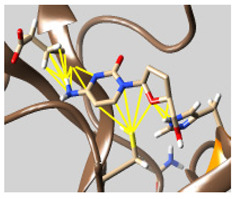
Telbivudine	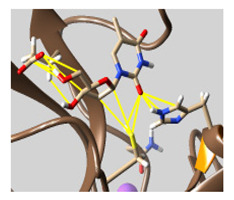	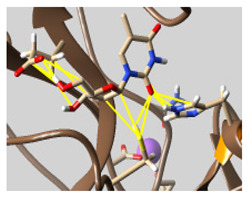	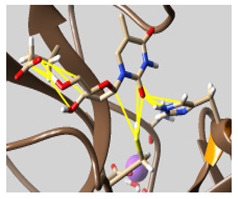
Tipiracil	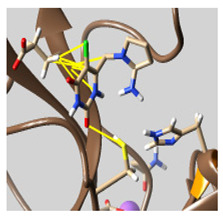	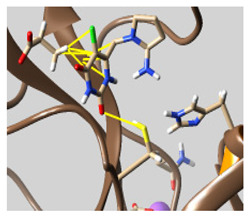	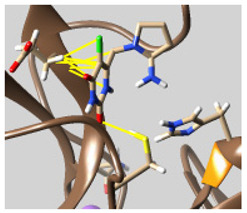
Citicoline	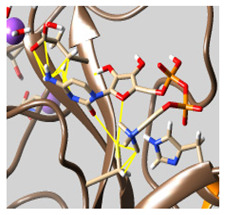	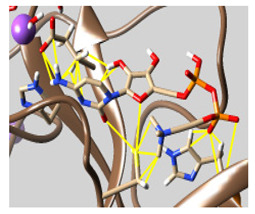	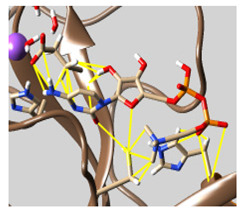
Uridine triacetate	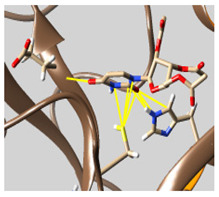	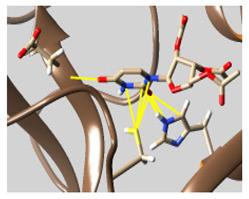	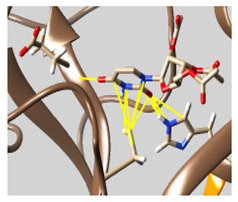

**Table 4:** The MMPBSA and MMGBSA data for the binding of pyrimidone containing-pharmaceuticals to 3CL^Pro^ of SARS-CoV-2.

3CL^pro^ Complex Type	−TΔS	E_vdw_	MMGBSA			MMPBSA		
			E_alac_	E_sol_	Δg (kcal/mol)	E_alac_	E_sol_	Δg (kcal/mol)
Uracil mustard	22.58	−23.76	−16.08	20.46	3.20	−0.80	0.94	−1.05
Cytarabine	22.39	−19.25	−33.50	28.39	−1.96	−1.67	1.09	2.55
Floxuridine	22.42	−24.54	0.00	3.70	1.58	0.00	0.39	−1.73
Trifluridine	22.96	−29.48	0.00	4.20	−2.32	0.00	0.41	−6.11
Stavudine	22.25	−24.41	−17.67	17.90	−1.94	−0.88	0.93	−2.12
Lamivudine	22.26	−20.58	−35.11	28.94	−4.50	−1.76	1.06	0.98
Zalcitabine	22.12	−24.34	−30.10	26.50	−5.81	−1.50	1.19	2.53
Telbivudine	22.48	−25.29	−46.88	42.53	−7.16	−2.34	1.61	−3.54
Tipiracil	22.54	−28.79	0.00	5.07	−1.18	0.00	0.44	−5.81
**Citicoline**	24.17	−54.50	0.00	4.80	**−25.53**	0.00	0.64	**−29.69**
**Uridine triacetate**	23.60	−32.98	−31.09	33.40	**−7.07**	−1.55	1.43	**−9.51**

## Data Availability

The data will be available upon request.

## Supplementary Materials

The following are available online, Table S1: The binding affinities of the pyrimidonic pharmaceuticals (group 3PPs) with 3-chymotrypsin-like protease (3CLpro), Table S2: The binding affinities of the pyrimidonic pharmaceuticals (group 2bPPs) with 3-chymotrypsin-like protease (3CLpro), Table S3: The binding affinities of the pyrimidonic pharmaceuticals (group 2aPPs) with 3-chymotrypsin-like protease (3CLpro), Table S4: The binding affinity of the pyrimidone containing-pharmaceuticals (group 1PCPs) with 3-chymotrypsinlike protease (3CLpro).
