# Supplementary material for: Molecular Docking and Dynamics Investigations for Identifying Potential Inhibitors of the 3-Chymotrypsin-like Protease of SARS-CoV-2: Repurposing of Approved Pyrimidonic Pharmaceuticals for COVID-19 Treatment
Source: Molecules. 2021 Dec 9;26(24):7458. doi: 10.3390/molecules26247458 (PMC8707611; doi:10.3390/molecules26247458)
Supplement: Supplementary file 1 [file molecules-26-07458-s001.zip › molecules-1424243-supplementary.pdf]

Supplementary material

Molecular docking and dynamics investigations for identifying potential inhibitors of the 3-chymotrypsin-like protease of SARS-CoV-2: repurposing of approved pyrimidonic pharmaceuticals for COVID-19 treatment

Amin Osman Elzupir*

College of Science, Deanship of Scientific Research, Imam Mohammad Ibn Saud Islamic University (IMSIU), Riyadh, Kingdom of Saudi Arabia.

* Correspondence: author email: aoalamalhuda@imamu.edu.sa

Table S1. The binding affinities of the pyrimidonic pharmaceuticals (group 3PPs) with 3-chymotrypsin-like protease ($3CL^{pro}$).

Pharmaceutical name	Binding percentage[a]	Score ± SD (kcal/mol)[b]	RMSD	Hydrogen bond (number of bonds/ number of conformations)	Van der Waals (number of bonds/ number of conformations)
Riboflavin	a. 22*<br>b. 11<br>c. 22<br>d. 22<br>e. 22<br>f. 44<br>All. 67	a. 6.4 ± 0.28<br>b. -6.1± 0.00<br>c. 6.4 ± 0.28<br>d. 6.4 ± 0.28<br>e. 6.4 ± 0.28<br>f. 6.3 ± 0.26	a. 22.37 - 25.40<br>b. 2.52 - 6.06<br>c. 22.37 - 25.40<br>d. 22.37 - 25.40<br>e. 22.37 - 25.40<br>f. 0.00 - 7.78	a. HIS163 (1/1), GLU 166 (3/1), PHE 140 (1/1)	a. HIS 163 (1/1), GLU 166 (3/1), PHE 140 (1/1)<br>b. LYS 5 (3/1)<br>c. HIS 164 (2/2), MET 49 (9/1)<br>d. GLN 189 (9/2), MET 165 (14/2)<br>e. GLY 143 (3/1), CYS 145 (4/2), SER 144 (1/1)<br>f. LEU 286 (32/4)
Alogliptin	a. 33<br>c. 22<br>d. 33<br>e. 33<br>f. 22<br>All. 56	a. -6.5 ± 0.44<br>c. -6.4 ± 0.49<br>d. -6.5 ± 0.44<br>e. -6.5 ± 0.44<br>f. 6.2 ± 0.00	a. 0.00 - 5.22<br>c. 2.02 - 5.22<br>d. 0.00 - 5.22<br>e. 0.00 - 5.22<br>f. 23.93 – 28.37	a. HIS 163 (2/2), GLU 166 (1/1)<br>e. GLY 143 (1/1)	a. HIS 163 (12/3), ASN 142 (27/2), PHE 140 (13/3), LEU 141 (7/3), GLU 166 (21/3)<br>c. HIS 164 (2/1), MET 49 (13/2)<br>d. GLN 189 (33/3), MET 165 (12/3)<br>e. GLY 143 (3/1), CYS 145 (9/3), SER 144 (4/2), HIS 41 (7/1)<br>f. LEU 286 (10/2)
Flavin mononucleotide	a. 33<br>c. 22<br>d. 33<br>e. 33<br>f. 33<br>All. 67	a. -6.8 ± 0.17<br>c. -6.9 ± 0.21<br>d. -6.8 ± 0.17<br>e. -6.8 ± 0.17<br>f. -6.4 ± 0.00	a. 0.00 - 7.56<br>c. 0.00 - 7.56<br>d. 0.00 - 7.56<br>e. 0.00 - 7.56<br>f. 21.94 - 31.82	a. ASN 142 (1/1), PHE 140 (2/2)<br>e. HIS 41 (1/1)	a. HIS 163 (15/2), ASN 142 (32/3), PHE 140 (9/2), LEU 141 (8/3), GLU 166 (47/3)<br>c. HIS 164 (1/1), MET 49 (19/2)<br>d. GLN 189 (11/3), MET 165 (13/2)<br>e. GLY 143 (1/1), CYS 145 (4/2), SER 144 (4/2), HIS 41 (26/3)<br>f. LEU 286 (29/3)

[a] Binding percentage was calculated based on the number of conformations attached to the active sites of the CL^{pro} (nine conformations in total).
[b] SD based on the other score energies of conformations.
* Alphabetical order indicates the type of active site involved in bonding: a. S1 site, b. S1 site from the other promotor, c. S2 site, d. S4 site, e. S'1 site, and f. SER 284, ALA 285, and LEU 286 residues. When letters are missing, this that means no interactions were observed at that site.

Table S1. continued

**Pharmaceutical name**	**Binding percentage**[a]	**Score ± SD (kcal/mol)**[b]	**RMSD**	**Hydrogen bond (number of bonds/ number of conformations)**	**Van der Waals (number of bonds/ number of conformations)**
Flavin adenine dinucleotide	a. 11[*]<br>b. 11<br>c. 11<br>d. 11<br>e. 11<br>f. 56<br>All. 67	a. -8.0 ± 0.00<br>b. -7.9 ± 0.00<br>c. -8.0 ± 0.00<br>d. -8.0 ± 0.00<br>e. -8.0 ± 0.00<br>f. -7.9 ± 0.15	a. 23.23 – 27.4<br>b. 4.15 – 6.59<br>c. 23.23 – 27.4<br>d. 23.23 – 27.4<br>e. 23.23 – 27.4<br>f. 0.00 - 11.47	a. ASN 142 (1/1)<br>e. GLY 143 (1/1)	a. HIS 163 (2/1), ASN 142 (15/1), PHE 140 (1/1), LEU 141 (5/1), GLU 166 (16/1)<br>b. LYS 5 (1/1)<br>c. MET 49 (14/1)<br>d. GLN 189 (13/1), MET 165 (2/1)<br>e. GLY 143 (2/1), CYS 145 (6/1), HIS 41 (2/1)<br>f. LEU 286 (47/5)
Trametinib	a. 11<br>b. 11<br>c. 11<br>d. 11<br>e. 11<br>f. 33<br>All. 56	a. -7.0 ± 0.00<br>b. -7.0 ± 0.00<br>c. -7.0 ± 0.00<br>d. -7.0 ± 0.00<br>e. -7.0 ± 0.00<br>f. -7.2 ± 0.15	a. 29.46 – 31.30<br>b. 20.22 – 22.34<br>c. 29.46 – 31.30<br>d. 29.46 – 31.30<br>e. 29.46 – 31.30<br>f. 16.55 - 20.17	-	a. HIS 163 (1/1), ASN 142 (11/1), PHE 140 (3/1), LEU 141 (5/1), GLU 166 (9/1)<br>b. PHE 3 (9/1), ARG 4 (8/1), LYS 5 (24/1)<br>c. MET 49 (10/1)<br>d. GLN 189 (12/1)<br>e. SER 144 (4/1), CYS 145 (4/1), HIS 41 (4/1)<br>f. ALA 285 (1/1), LEU 286 (35/3)
Dasabuvir	b. 33<br>f. 56<br>All. 56	b. -6.8 ± 0.21<br>f. -6.9 ± 0.22	b. 18.06 – 21.59<br>f. 18.06 - 22.94	b. LYS 5 (2/2)	b. ARG 4 (4/1), LYS 5 (8/3)<br>f. SER 284 (9/1), ALA 285 (9/3), LEU 286 (65/5)
Relugolix	b. 44<br>f. 78<br>All. 89	b. -6.8 ± 0.21<br>f. -6.9 ± 0.22	b. 18.06 – 21.59<br>f. 18.06 - 22.94	b. ALA 7 (1/1)	b. LYS 5 (46/4), MET 6 (5/1), ALA 7 (14/2)<br>f. SER 284 (11/1), LEU 286 (44/7)
Elagolix	f. 78<br>All. 89	f. -6.9 ± 0.22	f. 18.06 - 22.94	.	f. LEU 286 (5/1)
Sofosbuvir	a. - 11<br>b. - 22<br>c. - 11<br>d. - 11<br>e. - 11<br>f. 56<br>All. 67	a. -7.1 ± 0.00<br>b. -7.3 ± 0.07<br>c. -7.1 ± 0.00<br>d. -7.1 ± 0.00<br>e. -7.1 ± 0.00<br>f. -7.1 ± 0.14	a. 25.40 – 29.94<br>b. 0.00 – 9.62<br>c. 25.40 – 29.94<br>d. 25.40 – 29.94<br>e. 25.40 – 29.94<br>f. 0.00 – 9.62	a. ASN 142 (1/1), GLU 166 (1/1)<br>b. LYS 5 (1/1)	a. LEU 141 (2/1), ASN 142 (17/1), PHE 140 (2/1), HIS 163 (2/1), GLU 166 (12/1)<br>b. LYS 5 (7/2)<br>c. MET 49 (4/1)<br>d. MET 165 (3/1), GLN 189 (10/1)<br>e. HIS 41 (6/1), CYS 145 (4/1)<br>f. LEU 286 (36/5)

[a] Binding percentage was calculated based on the number of conformations attached to the active sites of the CL^{pro} (nine conformations in total).
[b] SD based on the other score energies of conformations.
* Alphabetical order indicates the type of active site involved in bonding: a. S1 site, b. S1 site from the other promotor, c. S2 site, d. S4 site, e. S'1 site, and f. SER 284, ALA 285, and LEU 286 residues. When letters are missing, this that means no interactions were observed at that site.

Table S2. The binding affinities of the pyrimidonic pharmaceuticals (group 2bPPs) with 3-chymotrypsin-like protease (3CL^{pro}).

Pharmaceutical name	Binding percentage[a]	Score ± SD (kcal/mol)[b]	RMSD	Hydrogen bond (number of bonds/ number of conformations)	van der Waals (distance) (number of bonds/ number of conformations)
Idoxuridine	a. 33*<br>c. 44<br>d. 33<br>e. 33<br>All. 44	a. 5.6 ± 0.26<br>c. 5.6 ± 0.26<br>d. 5.6 ± 0.26<br>e. 5.6 ± 0.26	a. 0.00 - 7.13<br>c. 0.0 – 15.11<br>d. 0.00 - 7.13<br>e. 0.00 - 7.13	a. HIS 163 (1/1), PHE 140 (1/1)<br>c. ARG 188 (1/1), HIS 164 (2/2)	a. ASN 142 (16/3), PHE 140 (4/1), HIS 163 (2/1), GLU 166 (3/1), LEU 141 (3/1).<br>c. ASP 187 (9/1), ARG 188 (19/1), TYR 54 (3/1), HIS 164 (5/3), MET 49 (17/3)<br>d. GLN 189 (10/2), MET 165 (1/1)<br>e. HIS 41 (27/3), CYS 145 (6/3)
Floxuridine	a. 44<br>c. 44<br>d. 44<br>e. 44<br>All. 56	a. 5.5± 0.13<br>c. 5.6± 0.25<br>d. 5.6± 0.25<br>e. 5.6± 0.19	a. 3.24 – 8.28<br>c. 0.00 – 8.28<br>d. 0.00 – 8.28<br>e. 0.00 – 8.28	a. ASN 142 (1/1), HIS 163 (2/2), GLU 166 (2/2), PHE 140 (1/1), LEU 141 (1/1)<br>e. HIS 41 (1/1)	a. GLU 166 (23/4), LEU 141 (6/2), PHE 140 (8/2), HIS 163 (5/2), ASN 142 (19/4)<br>c. MET 49 (9/2), HIS 164 (3/3)<br>d. MET 165 (5/3), GLN 189 (4/1)<br>e. GLY 143 (6/1), SER 144 (4/1), CYS 145 (5/3), HIS 41 (16/3)
Trifluridine	a. 44<br>c. 44<br>d. 44<br>e. 44<br>f. 11<br>All. 56	a. -6.03 ± 0.17<br>c. -6.03 ± 0.17<br>d. -6.03 ± 0.17<br>e. -6.03 ± 0.17<br>f. -5.7	a. 0.00 – 5.58<br>c. 0.00 – 5.58<br>d. 0.00 – 5.58<br>e. 0.00 – 5.58<br>f. 28.34 – 30.21	a. GLU 166 (2/2), ASN 142 (1/1).<br>c. HIS 164 (2/2)<br>e. GLY 143 (1/1)	a. GLU 166 (18/4), ASN 142 (15/3), HIS 163 (2/1), LEU 141 (2/1)<br>c. HIS 164 (10/3), MET 49 (17/4)<br>d. - MET 165 (19/4), GLN 189 (1/1).<br>e. CYS 145 (13/4), GLY 143 (10/2), HIS 41 (10/4)<br>f. SER 284 (9/1)
Telbivudine	a. 56<br>c. 44<br>d. 44<br>e. 56<br>f. 11<br>All. 67	a. -5.6 ± 0.38<br>c. -5.7 ± 0.42<br>d. -5.7 ± 0.42<br>e. -5.6 ± 0.38<br>f. -5.3	a. 0.00 – 7.50<br>c. 0.00 – 7.50<br>d. 0.00 – 7.50<br>e. 0.00 – 7.50<br>f. 22.85 – 24.17	a. ASN 142 (2/1), HIS 163 (3/3), GLU 166 (1/1), PHE 140 (2/2), LEU 141 (1/1)<br>c. HIS 164 (1/1)<br>d. GLN 189 (1/1)<br>e. HIS 41 (1/1)	a. GLU 166 (36/4), HIS 163 (11/3), PHE 140 (11/3), ASN 142 (27/5), LEU 141 (9/3)<br>c. HIS 164 (6/3), MET 49 (27/3)<br>d. GLN 189 (10/3), MET 165 (14/4),<br>e. CYS 145 (11/4), SER 144 (8/2), GLY 143 (3/1), HIS 41 (12/5)<br>f. LEU 286 (5/1)
Zidovdine	a. 44<br>c. 44<br>d. 33<br>e. 33<br>f. 22<br>All. 67	b. - 6.8 ± 0.21<br>f. -6.9 ± 0.22	a. 0.00 – 6.68<br>c. 0.00 – 6.68<br>d. 0.00 – 6.68<br>e. 0.00 – 6.68<br>f. 29.54 – 35.14	c. HIS 164 (1/1)<br>e. HIS 41 (1/1)	a. HIS 163 (11/2), GLU 166 (21/3), ASN 142 (18/3), LEU 141 (10/1), PHE 140 (3/1)<br>c. MET 49 (16/4), HIS 164 (2/2)<br>d. MET 165 (10/3), GLN 189 (1/1)<br>e. SER 144 (3/1), HIS 41 (18/2), GLY 143 (9/1), CYS 145 (7/2)<br>f. LEU 286 (5/2)
Stavudine	a. 44*<br>c. 56<br>d. 44<br>e. 56<br>All. 56	a. -5.6 ± 0.28<br>c. -5.6 ± 0.28<br>d. -5.6 ± 0.28<br>e. -5.6 ± 0.28	a. 27.12 – 32.34<br>c. 27.12 – 35.03<br>d. 27.12 – 32.34<br>e. 27.12 – 35.03	a. GLU 166 (1/1)	a. ASN 142 (13/3), GLU 166 (14/4), HIS 163 (2/1), LEU 141 (1/1)<br>c. HIS 164 (6/3), MET 49 (20/5)<br>d. - MET 165 (12/4), GLN 189 (1/1)<br>e. HIS 41 (39/5), GLY 143 (9/2), CYS 145 (7/3)

[a] Binding percentage was calculated based on the number of conformations attached to the active sites of the CL^{pro} (nine conformations in total).
[b] SD based on the other score energies of conformations.
* Alphabetical order indicates the type of active site involved in bonding: a. S1 site, b. S1 site from the other promotor, c. S2 site, d. S4 site, e. S'1 site, and f. SER 284, ALA 285, and LEU 286 residues. When letters are missing, this that means no interactions were observed at that site.

Table S2. continued

Pharmaceutical name	Binding percentage[a]	Score ± SD (kcal/mol)[b]	RMSD	Hydrogen bond (number of bonds/ number of conformations)	van der Waals (distance) (number of bonds/ number of conformations)
Brivudine	a. 22<br>b. 22<br>c. 11<br>d. 22<br>e. 22<br>f. 11<br>All. 44	a. -5.8 ± 0.35<br>b. -5.6 ± 0.07<br>c. -6.0<br>d. -5.8 ± 0.35<br>e. -5.8 ± 0.35<br>f. -5.5	a. 0.00 – 5.84<br>b. 20.43 – 23.62<br>c. 0.00 – 0.00<br>d. 0.00 – 5.84<br>e. 0.00 – 5.84<br>f. 21.49 – 23.62	a. HIS 163 (1/1), GLU 166 (3/2), LEU 141(1/1)	a. HIS 163 (7/2), GLU 166 (39/2), LEU 141 (9/2), PHE 140 (11/2), ASN 142 (13/2)<br>b. LYS 5 (3/2)<br>c. MET 49 (4/1)<br>d. LEU 167 (2/1), GLN 189 (5/1), MET 165 (2/1)<br>e. CYS 145 (4/2), SER 144 (2/1)<br>f. LEU 286 (1/1)
Tegafur	a. 11<br>c. 11<br>d. 11<br>e. 22<br>All. 22	a. -5.3<br>c. -5.2<br>d. -5.3<br>e. -5.3 ± 0.07	a. 26.33 – 27.44<br>c. 30.42 – 31.53<br>d. 26.33 – 27.44<br>e. 26.33 – 31.53	a. PHE 140 (1/1), GLU 166 (1/1), HIS 163(1/1)	a. PHE 140 (2/1), GLU 166 (12/1), ASN 142 (3/1), HIS 163 (2/1), LEU 141 (4/1)<br>c. MET 49 (5/1)<br>d. MET 165 (5/1)<br>e. HIS 41 (7/1), CYS 145 (3/2)
Uridine triacetate	a. 56<br>c. 56<br>d. 56<br>e. 56<br>f. 11<br>All. 67	a. -6.2 ± 0.26<br>c. -6.2 ± 0.26<br>d. -6.2 ± 0.26<br>e. -6.2 ± 0.26<br>f. -6.4	a. 0.00 – 6.79<br>c. 0.00 – 6.79<br>d. 0.00 – 6.79<br>e. 0.00 – 6.79<br>f. 24.97 – 28.01	a. HIS 163 (2/2), GLU 166 (3/2)<br>e. HIS 41 (1/1)	a. HIS 163 (5/2), GLU 166 (31/5), ASN 142 (30/5), PHE 140 (4/2), LEU 141 (6/2)<br>c. MET 49 (18/5), HIS 164 (5/2)<br>d. GLN 189 (15/4), MET 165 (18/4)<br>e. CYS 145 (16/5), HIS 41 (35/5), SER 144 (4/2), GLY 143 (2/1)<br>f. LEU 286 (1/1)
Tipiracil	a. 56<br>b. 11<br>c. 44<br>d. 44<br>e. 44<br>f. 11<br>All. 67	a. -5.8 ± 0.16<br>b. -5.7<br>c. -5.9 ± 0.17<br>d. -5.8 ± 0.08<br>e. -5.9 ± 0.17<br>f. -5.7	a. 26.28 – 29.69<br>b. 19.12 – 20.08<br>c. 26.28 – 29.69<br>d. 26.67 – 29.69<br>e. 26.28 – 29.69<br>f. 19.12 – 20.08	a. HIS 163 (2/2), GLU 166 (2/1), PHE 140 (2/2), ASN 142 (1/1)<br>b. LYS 5 (1/1)<br>c. HIS 164 (1/1)<br>e. GLY 143 (1/1)	a. HIS 163 (14/3), GLU 166 (40/4), PHE 140 (8/3), ASN 142 (22/4), LEU 141 (10/3)<br>b. PHE 3 (6/1), LYS 5 (9/1), ARG 4 (5/1)<br>c. HIS 164 (8/4), MET 49 (22/3)<br>d. MET 165 (9/3), GLN 189 (13/3)<br>e. GLY 143 (7/2), HIS 41 (26/4), SER 144(2/1), CYS 145 (13/4)<br>f. SER 284 (7/1), LEU 286 (2/1)
Enprofylline	a. 33*<br>c. 33<br>d. 22<br>e. 33<br>f. 11<br>All. 44	a. 5.0 ± 0.17<br>c. -5.0 ± 0.17<br>d. -5.0 ± 0.21<br>e. -5.0 ± 0.17<br>f. -4.8	a. 0.00 – 9.13<br>c. 0.00 – 9.13<br>d. 0.00 – 4.75<br>e. 0.00 – 9.13<br>f. 27.78 – 28.50	a. HIS 163 (1/1), GLU 166 (1/1), PHE 140 (1/1)<br>c. HIS 164 (1/1)	a. GLU 166 (11/2), HIS 163 (6/2), ASN 142 (14/3), LEU 141 (9/2), PHE 140 (4/2)<br>c. MET 49 (3/2), HIS 164 (3/2)<br>d. MET 165 (6/2)<br>e. SER 144 (2/1), HIS 41 (15/2), CYS 145 (6/2)<br>f. LEU 286 (2/1)

[a] Binding percentage was calculated based on the number of conformations attached to the active sites of the CL^{pro} (nine conformations in total).
[b] SD based on the other score energies of conformations.
* Alphabetical order indicates the type of active site involved in bonding: a. S1 site, b. S1 site from the other promotor, c. S2 site, d. S4 site, e. S'1 site, and f. SER 284, ALA 285, and LEU 286 residues. When letters are missing, this that means no interactions were observed at that site.

Table S3. The binding affinities of the pyrimidonic pharmaceuticals (group 2aPPs) with 3-chymotrypsin-like protease (3CL^{pro}).

Pharmaceutical name	Binding percentage[a]	Score ± SD (kcal/mol)[b]	RMSD	Hydrogen bond (number of bonds/ number of conformations)	van der Waals (distance) (number of bonds/ number of conformations)
Gemcitabine	a. 33*<br>c. 33<br>d. 33<br>e. 33<br>f. 22<br>All. 56	a. -5.9 ± 0.17<br>c. -5.9 ± 0.17<br>d. -5.9 ± 0.17<br>e. -5.9 ± 0.17<br>f. -5.6 ± 0.0	a. 27.34 – 31.14<br>c. 27.34 – 31.14<br>d. 27.34 – 31.14<br>e. 27.34 – 31.14<br>f. 18.30 – 22.26	a. GLU 166 (2/ 2), ASN 142 (1/1), PHE 140 (1/1), HIS 163 (1/1)<br>e. SER 144 (1/1)	a. GLU 166 (31/3), ASN 142 (11/2), PHE 140 (7/2), HIS 163 (8/2), LEU 141 (10/2)<br>c. MET 49 (12/2), HIS 164 (3/2)<br>d. MET 165 (13/3), GLN 189 (1/1)<br>e. SER 144 (4/1), CYS 145 (4/3), HIS 41 (2/1)<br>f. LEU 286 (8/2)
Lamivudine	a. 56<br>c. 44<br>d. 44<br>e. 56<br>f. 11<br>All. 67	a. -5.4 ± 0.24<br>c. -5.4 ± 0.28<br>d. -5.4 ± 0.25<br>e. -5.4 ± 0.24<br>f. -5.2	a. 0.00 – 4.77<br>c. 0.00 – 3.38<br>d. 0.00 – 4.77<br>e. 0.00 – 4.77<br>f. 26.28 – 28.62	a. HIS 163 (3/3), ASN 142 (1/1), PHE 140 (3/3), LEU 141 (2/2), GLU 166 (2/2)<br>d. GLN 189 (1/1)<br>e. SER 144 (2/2)	a. HIS 163 (26/5), ASN 142 (19/2), PHE 140 (31/5), LEU 141 (15/5), GLU 166 (46/5)<br>c. MET 49 (12/4), HIS 164 (1/1)<br>d. GLN 189 (12/2), MET 165 (10/4), LEU 167 (1/1)<br>e. SER 144 (18/4), HIS 41 (5/2), CYS 145 (7/3)<br>f. LEU 286 (1/1)
Emtricitabine	a. 22<br>b. 22<br>c. 11<br>d. 22<br>e. 22<br>All. 44	a. -5.5 ± 0.14<br>b. -5.2 ± 0.64<br>c. -5.6<br>d. -5.5 ± 0.14<br>e. -5.5 ± 0.14	a. 21.05 – 23.08<br>b. 0.00 – 12.76<br>c. 22.10 – 23.08<br>d. 21.05 – 23.08<br>e. 21.05 – 23.08	a. PHE 140 (2/2), GLU 166 (1/1)<br>b. LYS 5 (2/1)	a. PHE 140 (10/2), GLU 166 (21/2), HIS 163 (7/2), LEU 141 (7/2), ASN 142 (1/1)<br>b. LYS 5 (14/2), PHE 3 (8/1), ARG 4 (3/1)<br>c. MET 49 (4/1), HIS 164 (1/1)<br>d. MET 165 (6/2), GLN 189 (4/1), LEU 167 (1/1)<br>e. SER 144 (4/1), CYS 145 (2/1)
Zalcitabine	a. 44<br>b. 11<br>c. 33<br>d. 44<br>e. 44<br>f. 22<br>All. 67	a. -5.5 ± 0.29<br>b. -5.1<br>c. -5.4 ± 0.35<br>d. -5.5 ± 0.29<br>e. -5.5 ± 0.29<br>f. -5.1 ± 0.00	a. 0.00 – 6.39<br>b. 28.02 – 29.48<br>c. 0.00 – 6.39<br>d. 0.00 – 6.39<br>e. 0.00 – 6.39<br>f. 28.02 – 31.88	a. PHE 140 (3/3), LEU 141 (1/1), GLU 166 (3/3), ASN 142 (1/1)<br>c. GLN 189 (1/1)	a. HIS 163 (15/3), PHE 140 (16/3), LEU 141 (9/3), GLU 166 (25/4), ASN 142 (11/3)<br>b. LYS 5 (6/1), ARG 4 (12/1), PHE 3 (4/1)<br>c. MET 49 (16/2), HIS 164 (5/3)<br>d. GLN 189 (6/2), MET 165 (15/4)<br>e. SER 144 (7/2), HIS 41 (13/2), CYS 145 (6/3)<br>f. SER 284 (4/1), LEU 286 (4/1)
Cytarabine	a. 67<br>c. 67<br>d. 67<br>e. 67<br>All. 67	a. -5.4 ± 0.26<br>c. -5.4 ± 0.26<br>d. -5.4 ± 0.26<br>e. -5.4 ± 0.26	a. 0.00 – 6.07<br>c. 0.00 – 6.07<br>d. 0.00 – 6.07<br>e. 0.00 – 6.07	a. GLU 166 (3/3), LEU 141 (2/2), HIS 163 (1/1), PHE 140 (2/2), ASN 142 (1/1)<br>c. HIS 164 (2/2)<br>d. GLN 189 (1/1)<br>e. GLY 143 (1/1)	a. GLU 166 (44/5), LEU 141 (18/3), HIS 163 (18/4), PHE 140 (19/3), ASN 142 (27/6)<br>c. HIS 164 (10/4), MET 49 (20/6)<br>d. MET 165 (28/4), GLN 189 (4/2)<br>e. GLY 143 (11/2), SER 144 (8/2), CYS 145 (15/6), HIS 41 (21/5)
Capecitabine	a. 11<br>c. 22<br>d. 11<br>e. 11<br>f. 33<br>All. 56	a. -6.1<br>c. 6.0 ± 0.41<br>d. -6.1<br>e. -6.1<br>f. -6.1 ± 0.29	a. 26.06 – 30.29<br>c. 26.06 – 31.05<br>d. 26.06 – 30.29<br>e. 26.06 – 30.29<br>f. 0.00 – 3.68	-	a. GLU 166 (9/1), HIS 163 (1/1)<br>c. ARG 188 (7/1), MET 49 (10/1), TYR 54 (6/1), HIS 164 (1/1)<br>d. MET 165 (3/1), GLN 189 (1/1)<br>e. HIS 41 (3/1), CYS 145 (2/1)<br>f. LEU 286 (10/3)

[a] Binding percentage was calculated based on the number of conformations attached to the active sites of the CL^{pro} (nine conformations in total).
[b] SD based on the other score energies of conformations.
* Alphabetical order indicates the type of active site involved in bonding: a. S1 site, b. S1 site from the other promotor, c. S2 site, d. S4 site, e. S'1 site, and f. SER 284, ALA 285, and LEU 286 residues. When letters are missing, this that means no interactions were observed at that site.

Table S3. continued

**Pharmaceutical name**	**Binding percentage**[a]	**Score ± SD (kcal/mol)**[b]	**RMSD**	**Hydrogen bond (number of bonds/ number of conformations)**	**van der Waals (distance) (number of bonds/ number of conformations)**
Sulfacytine	a. 22<br>b. 11<br>c. 22<br>d. 22<br>e. 22<br>f. 22<br>All. 56	a. -6.3 ± 0.07<br>b. -6.2<br>c. -6.3 ± 0.07<br>d. -6.3 ± 0.07<br>e. -6.3 ± 0.07<br>f. -6.4 ± 0.21	a. 28.69 – 31.24<br>b. 18.44 – 20.59<br>c. 28.69 – 31.24<br>d. 28.69 – 31.24<br>e. 28.69 – 31.24<br>f. 19.75 – 22.63	a. ASN 142 (1/1)<br>e. GLY 143 (1/1), HIS 41 (1/1)	a. ASN 142 (11/2), GLU 166 (5/2), HIS 163 (2/1)<br>b. LYS 5 (3/1)<br>c. MET 49 (4/2), HIS 164 (1/1)<br>d. GLN 189 (2/1), MET 165 (3/2)<br>e. GLY 143 (4/1), HIS 41 (13/2), CYS 145 (7/2)<br>f. LEU 286 (11/2)
Citicoline	a. 56<br>c. 56<br>d. 56<br>e. 56<br>All. 56	a. - 7.0 ± 0.19<br>c. - 7.0 ± 0.19<br>d. - 7.0 ± 0.19<br>e. - 7.0 ± 0.19	a. 0.00 – 8.01<br>c. 0.00 – 8.01<br>d. 0.00 – 8.01<br>e. 0.00 – 8.01	a. PHE 140 (2/2), GLU 166 (4/4), HIS 163 (3/3), ASN 142 (2/1), LEU 141 (1/1)<br>e. SER 144 (1/1)	a. PHE 140 (24/5), GLU 166 (57/5), HIS 163 (21/5), ASN 142 (34/5), LEU 141 (21/5)<br>c. MET 49 (23/5), HIS 164 (1/1)<br>d. MET 165 (29/5), GLN 189 (6/3)<br>e. SER 144 (12/3), GLY 143 (4/1), CYS 145 (9/5), HIS 41 (21/5)

[a] Binding percentage was calculated based on the number of conformations attached to the active sites of the CL^{pro} (nine conformations in total).
[b] SD based on the other score energies of conformations.
* Alphabetical order indicates the type of active site involved in bonding: a. S1 site, b. S1 site from the other promotor, c. S2 site, d. S4 site, e. S'1 site, and f. SER 284, ALA 285, and LEU 286 residues. When letters are missing, this that means no interactions were observed at that site.

Table S4. The binding affinity of the pyrimidone containing-pharmaceuticals (group 1PCPs) with 3-chymotrypsin-like protease (3CL^{pro}).

Pharmaceutical name	Binding percentage	Score ± SD (kcal/mol)	RMSD	Hydrogen bond	Vander Val (distance)
Cidofovir	a. 56<br>c. 44<br>d. 44<br>e. 56<br>All. 56	a. -5.2 ± 0.25<br>c. -5.2 ± 0.29<br>d. -5.2 ± 0.28<br>e. -5.2 ± 0.25	a. 0.00 – 6.27<br>c. 0.00 – 6.27<br>d. 0.00 – 6.27<br>e. 0.00 – 6.27	a. HIS 163 (1/1), LEU 141 (1/1), GLU 166 (4/4), PHE 140 (1/1), ASN 142 (1/1)<br>c. HIS 164 (1/1)<br>d. GLN 189 (1/1)<br>e. SER 144 (1/1)	a. HIS 163 (21/5), LEU 141 (15/4), GLU 166 (36/5), PHE 140 (8/4), ASN 142 (20/5)<br>c. MET 49 (12/3), HIS 164 (10/4)<br>d. MET 165 (13/4), GLN 189 (5/1)<br>e. SER 144 (13/4), CYS 145 (19/5), HIS 41 (24/5), GLY 143 (4/2)
5-Fluorouracil	a. 11<br>b. 33<br>e. 11<br>f. 33<br>All. 44	a. -4.2<br>b. -4.4 ± 0.12<br>e. -4.2<br>f. -4.4 ± 0.12	a. 45.32 – 45.82<br>b. 21.72 – 23.15<br>e. 45.32 – 45.82<br>f. 21.72 – 23.15	a. PHE 140 (1/1), HIS 163 (1/1)<br>b. LYS 5 (2/2)	a. HIS 163 (5/1), LEU 141 (6/1), PHE 140 (3/1), ASN 142 (3/1), GLU 166 (6/1)<br>b. LYS 5 (10/3), PHE 3 (5/3), ARG 4 (9/3)<br>e. SER 144 (2/1)<br>f. SER 284 (9/3)
Uracil mustard	a. 100<br>b. -<br>c. 89<br>d. 100<br>e. 100<br>f. -<br>All. 100	a. -4.6 ± 0.14<br>b. -<br>c. -4.6 ± 0.14<br>d. -4.6 ± 0.14<br>e. -4.6 ± 0.14	a. 0.00 – 7.13<br>b. -<br>c. 0.00 – 7.13<br>d. 0.00 – 7.13<br>e. 0.00 – 7.13	a. HIS 163 (5/5), GLU 166 (3/3), LEU 141 (3/3), ASN 142 (1/1)	a. HIS 163 (31/5), GLU 166 (40/8), LEU 141 (19/5), ASN 142 (50/7), PHE 140 (8/5)<br>c. MET 49 (52/7), HIS 164 (12/4)<br>d. GLN 189 (32/9), MET 165 (28/8)<br>e. HIS 41 (53/9), SER 144 (10/5), GLY 143 (4/1), CYS 145 (28/9)
Flucytosine	a. 11<br>b. 22<br>d. 11<br>f. 33<br>All. 44	a. -4.4<br>b. -4.3 ± 0.07<br>d. -4.4<br>f. -4.3 ± 0.06	a. 23.3 – 23.81<br>b. 32.05 – 33.89<br>d. 23.3 – 23.81<br>f. 32.05 – 36.93	a. HIS 163 (1/1), PHE 140 (1/1)	a. HIS 163 (3/1), PHE 140 (5/1), GLU 166 (7/1), ASN 142 (9/1), LEU 141 (5/1)<br>b. PHE 3 (7/1), LYS 5 (5/1), ARG 4 (6/1)<br>d. MET 165 (1/1)<br>f. SER 284 (13/2), LEU 286 (2/1)

[a] Binding percentage was calculated based on the number of conformations attached to the active sites of the CL^{pro} (nine conformations in total).
[b] SD based on the other score energies of conformations.
* Alphabetical order indicates the type of active site involved in bonding: a. S1 site, b. S1 site from the other promotor, c. S2 site, d. S4 site, e. S'1 site, and f. SER 284, ALA 285, and LEU 286 residues. When letters are missing, this that means no interactions were observed at that site.
